# Regarding the study by Packer *et al*

**DOI:** 10.1093/jhps/hnab011

**Published:** 2021-05-29

**Authors:** Onur Hapa

**Affiliations:** Department of Orthopedic Surgery, Dokuz Eylul University, Izmir 035340, Turkey

To the Editor

I read with interest the article ‘Capsular thinning on magnetic resonance arthrography is associated with intra-operative hip joint laxity in women’ by Packer *et al*. [1] in the July 2020 issue of Journal of Hip Preservation.

On page 300 of the article at Fig. 1, the authors describe thickness of the hip joint capsule at the left image and the width of the anterior hip joint recess at the right image. When we compare this study with similar study by Magerkurth *et al*. [2] which they described the similar measurements at Fig. 1. of their study (thickness of joint capsule and width of joint recess lateral to zona orbicularis) it seems that figure legends are stirred up at the present study that left image shows the joint recess and right one the hip capsule.

## CONFLICT OF INTEREST STATEMENT

None declared. 
